# Chemotherapy-Induced Jejunal Perforations as an Atypical Presentation of Neutropenic Enterocolitis in an Acute Leukemia Patient

**DOI:** 10.7759/cureus.71636

**Published:** 2024-10-16

**Authors:** Zlatan Zvizdic, Asmir Jonuzi, Lejla Pilav, Irmina Sefic Pasic, Semir Vranić

**Affiliations:** 1 Department of Pediatric Surgery, Clinical Center University of Sarajevo, Sarajevo, BIH; 2 Department of Pediatrics, Clinical Center University of Sarajevo, Sarajevo, BIH; 3 Department of Radiology, Clinical Center University of Sarajevo, Sarajevo, BIH; 4 Department of Pathology, College of Medicine, Qatar University, Doha, QAT

**Keywords:** acute myelogenous leukemia, chemotherapy, children, gastrointestinal tract complications, neutropenic enterocolitis

## Abstract

Neutropenic enterocolitis (NE) is a potentially life-threatening condition, primarily affecting neutropenic patients with hematologic malignancies. The clinical manifestations of NE in patients receiving antineoplastic drugs range from fever, diarrhea, nausea, vomiting, and abdominal pain to intestinal perforation and shock. We report the case of a 12-year-old boy with acute myelogenous leukemia, undergoing chemotherapy, who presented with an atypical case of NE. Due to numerous jejunal perforations and severe rectal bleeding, he experienced abdominal distension without any accompanying tenderness and the unexpected rapid onset of shock. Surgery was performed, and his postoperative course was uneventful. However, seven days later, Pseudomonas aeruginosa-induced sepsis made his condition rapidly worse due to severe neutropenia and thrombocytopenia. Despite intensive supportive therapy, the patient unfortunately passed away. NE remains a life-threatening complication in pediatric immunosuppressed leukemic patients. A high index of suspicion, prompt diagnosis, aggressive treatment with broad-spectrum antibiotics, and correction of fluid-electrolyte imbalances are crucial in reducing morbidity and mortality.

## Introduction

Acute leukemia is the most common pediatric malignancy worldwide, corresponding to one-third of all childhood malignancies [1]. While ~80% of pediatric leukemias are acute lymphoblastic leukemia, acute myeloid leukemia (AML) accounts for the remaining 20%. Outcomes for pediatric AML have improved significantly over the past decades, with current survival rates as high as 70% due to the introduction of intensive myelosuppressive chemotherapy and stem cell transplantation in a certain subgroup of patients [2]. However, side effects of therapy are significant issues that need to be seriously addressed. Chemotherapy-induced neutropenic enterocolitis (NE) is seen in ~6-12% of pediatric cancer patients. It is a potentially life-threatening condition with substantial morbidity and mortality [3]. Although the pathogenesis of NE is not completely understood, it is considered that chemotherapy-induced mucositis or intestinal distension and necrosis, along with neutropenia and the immunocompromised state of the afflicted patients, are responsible for developing this condition [4]. A chemotherapeutic agent cytosine arabinoside (cytarabine) is particularly associated with the development of NE [4]. The most common symptoms of NE are abdominal pain, diarrhea, and fever, followed by nausea, vomiting, and abdominal distension [4]. As the condition progresses, localized and then generalized peritonitis and sepsis appear. NE usually affects the terminal ileum, cecum, or ascending colon. In rare instances, NE may involve more proximal or distal bowel.

Herein, we report a rare case of NE presenting with multiple jejunal perforations and severe lower intestinal bleeding following chemotherapy in a child with AML.

## Case presentation

**Figure 1:**
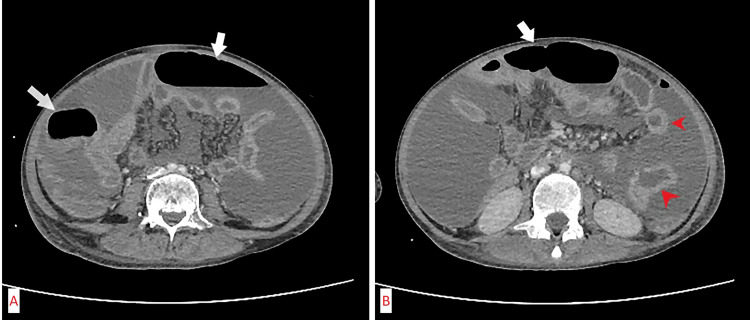
(A) Axial post-contrast CT scan of the abdomen showing signs of pneumoperitoneum (white arrows) and (B) axial CT scan of the abdomen showing postcontrast enhancement of small bowel wall as a sign of inflammation (pointed red arrows), free air in the abdomen (white arrow) after bowel perforation.

A 12-year-old boy was referred from the regional hospital to our tertiary pediatric facility due to massive pleural and pericardial effusions. His complaints started a month earlier with back and abdominal pain. Based on the results of sternal puncture, cytomorphology, cytogenetics, and immunophenotyping, a diagnosis of AML, FAB classification M0/M1, was made. After open-tunneled insertion of the Hickman central venous catheter via the right internal jugular vein, the AML BFM 2019 protocol was initiated for the patient. He received induction intrathecal chemotherapy with cytarabine 30 mg, methotrexate 12 mg, and prednisolone 10 mg. The therapy was continued with cytarabine in a dose of 100 mg/m^2^ daily by continuous IV infusion (days 1-3) and then in a dose of 100 mg/m^2^ daily IV every 12 hours (days 3-8), L-DNR-replacement doxorubicin in a dose of 50mg/m^2^/day, 3rd, 5th, 7th day, infusion for 120 min, and etoposide in a dose of 150mg/m^2^/day, (Days 6-8), infusion for 60 min. Induction was uneventful except on the last day (day 8) when the patient developed a fever, skin rash, diarrhea, vomiting, and conjunctival suffusion. The patient received platelet transfusions, concentrated filtered erythrocytes, antibiotics, and granulocyte colony-stimulating factor. He was also treated with nasogastric decompression, bowel rest, and total parental nutrition. However, despite supportive therapy, the patient had neutropenia (neutrophil count declined below 500 cells/mm^3^). The patient did not recover, complaining of anorexia, abdominal distension, severe pain, and rectal bleeding. Blood cultures revealed the growth of *Pseudomonas aeruginosa*, which was treated with meropenem. Physical examination revealed a distended abdomen, diffuse tenderness, and guarding. Abdominal ultrasonography showed thickened and dilatated small bowel loops with a diameter above 45 mm, no peristalsis, and intraperitoneal free fluid. Contrast-enhanced computed tomography (CT) showed that there was free air in the abdomen, the walls were 7 mm thick, and the dilated small intestine was inflamed (Figures 1A, 1B).

**Figure 2:**
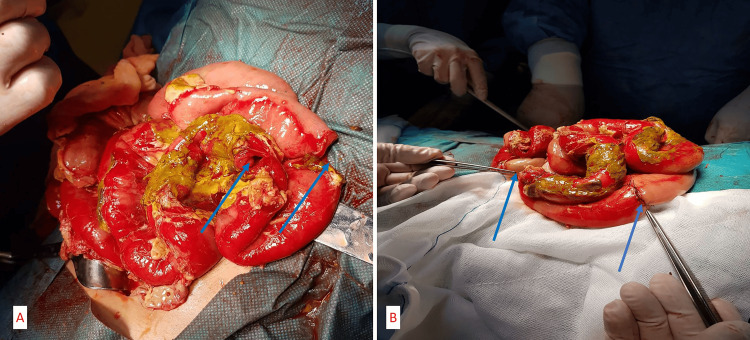
(A) Intraoperative images showing the perforations of the jejunum (blue arrows) and (B) functional end-to-end jejunal anastomoses, created by an anatomic side-to-side technique (blue arrows).

The patient then underwent emergency surgery. The surgical view on laparotomy revealed two perforations on the antimesenteric side of the dilatated jejunum at approximately 40 and 50 cm aboral from the ligament of Treitz, leading to diffuse fibrinopurulent peritonitis with turbid ascitic fluid exceeding 6000 mL in volume (Figure 2A).

A wedge resection of perforation sites and anastomoses was done (Figure 2B). The patient’s postoperative period was uneventful. On the 12th postoperative day, a sternal puncture was performed, and flow cytometric monitoring of minimal residual disease identified up to 0.5% of the blast population in a sample of unseparated bone marrow cells. Considering the good general condition of the patient along with normal hematologic and biochemical markers, the continuation of the chemotherapy according to the protocol AML-BFM 2019 was recommended. The patient got the second cycle of HAM, which included 40 mg of cytarabine injected into the spinal cord. They then got six doses of 3 g/m^2^ cytarabine through an IV over three hours every 12 hours. Additionally, mitoxantrone was given at 10 mg/m^2^/day as a 30-minute infusion on days 3 and 4. However, the patient became febrile seven days after receiving therapy. The next day, the patient had hypotension, reduced diuresis, and tachycardia with a decrease in leukocytes (0.1 × 103/mm^3^) and platelets (4 × 103/mm^3^), while C-reactive protein was 150.5 mg/L. A perianal hematoma was also noticed. The patient received thrombocytes and filtered red blood cell concentrate transfusions. The day after, the patient's condition deteriorated rapidly and progressively due to cardiac arrest. The patient received cardiopulmonary resuscitation and mechanical ventilation. Despite all measures taken, death occurred on the same day. The blood culture taken before death showed recurrent *Pseudomonas aeruginosa* infection, which was sensitive to administered antibiotics (meropenem).

## Discussion

This case report highlights an unusual presentation of NE as jejunal perforations and severe lower intestinal bleeding in a child treated with chemotherapy due to AML. It is also of utmost importance to understand the relationship between cytarabine therapy and NE to improve patient outcomes. Chemotherapy-induced neutropenia is a frequently encountered complication of intensive chemotherapy in oncology practice. NE is usually seen 7-10 days after the completion of chemotherapy, during the neutropenic phase [5]. This observation of the time of appearance of NE was confirmed in our case as NE developed on the eighth day of induction chemotherapy and continued to progress in severity. The range of clinical manifestations is broad, encompassing self-limiting disease to rapidly progressing conditions that ultimately result in death. An increased index of suspicion, prompt diagnosis, vigorous therapy with broad-spectrum antibiotics, and correction of fluid-electrolyte imbalances might enhance the clinical condition and avert unfavorable consequences like sepsis, multiple organ failure, and fatality. Clinical symptoms of NE are highly variable, and therefore, the diagnosis of this condition is widely considered to be clinical as no specific criteria exist to date. The most commonly presenting symptoms are fever, abdominal pain, and tenderness, diarrhea or constipation, nausea, and vomiting. Our patient had a fever, skin rash, diarrhea, vomiting, and conjunctival suffusion, while the abdominal pain and tenderness occurred somewhat later, probably due to the masking of the symptoms with administered corticosteroids. It is well known that chemotherapy-induced myelosuppression can complicate hematologic malignancies by causing severe hemorrhage, including gastrointestinal. In our case, rectal bleeding was probably the result of the intestinal wall injury at the site of perforations.

Some chemotherapeutic drugs that have been shown to cause NE are corticosteroids, cytarabine, vincristine, cyclophosphamide, irinotecan, cisplatin, and daunomycin [6]. In our case, cytarabine was the drug likely responsible for the development of NE. The way cytarabine damages the intestines is likely by starting up oxidative damage, the immune system, and apoptosis [7]. Literature data indicate that NE occurs after the first week after initiation of chemotherapy, corresponding to the neutrophil count nadir, similar to our case in which NE developed eight days after initiating chemotherapy. Since neutrophils are an important part of acute inflammation and the body's defense against bacterial infections, a lack of these cells, along with cytotoxic agents that directly damage the intestinal mucosa, allows bacteria to grow and enter through the bowel wall [8]. Endotoxin production further causes the bowel wall to become progressively ischemic, ulcerated, and necrotic [8]. Intestinal involvement in NE includes most commonly terminal ileum, cecum, and ascending colon, probably due to poor vascularization. However, any bowel segment, including the small intestine, may be involved. Interestingly, our patient had jejunal involvement in NE with two perforations, a far rarer localization than ileal or right colonic perforations, with only a few cases reported in the literature [9,10]. Bowel perforation linked to cytotoxic drugs, especially when corticosteroids are given at the same time and cause symptoms that look like the underlying condition, can have a strange clinical presentation. It may take longer to diagnose, which can lead to higher rates of illness and death, as in our case.

## Conclusions

In conclusion, NE is a life-threatening digestive complication associated with neutropenia in pediatric immunosuppressed leukemic patients. Early recognition, quick diagnosis, strong treatment with broad-spectrum antibiotics, and fixing fluid and electrolyte imbalances are some of the most important things that can lower morbidity and mortality by 20% to 50%. Although the terminal ileum, caecum, and ascending colon are most often involved in the process, any bowel segment including the jejunum may be involved. Achieving a good balance between effective AML treatment and minimizing side effects demands a nuanced, patient-focused approach, often involving dose modifications and supportive therapies customized to each patient's needs.
